# The hidden link between iron deficiency and celiac disease: a clinical perspective

**DOI:** 10.3389/fimmu.2026.1744005

**Published:** 2026-02-19

**Authors:** Laura Tarancon-Diez, Guillermo Perez-Cabeza, Pilar Sanchez-Mingo, Pedro Crespo-de la Rosa, Rita-Maria Garcia, Marianela Iriarte-Gahete, Yolanda M. Pacheco, Manuel Leal

**Affiliations:** 1 Pediatric Infections Group, Health Research Institute Gregorio Marañón (IiSGM), Madrid, Spain; 2 Centro de Investigación Biomédica en Red de Enfermedades Infecciosas (CIBER-INFEC), Instituto de Salud Carlos III (ISCIII), Madrid, Spain; 3 Emergency Department, Hospital Viamed Santa Ángela de la Cruz, Seville, Spain; 4 Eurofins Hospital Viamed Santa Ángela de la Cruz, Seville, Spain; 5 Servicio de Anatomia Patologica (SEVISTON), Hospital Viamed Santa Ángela de la Cruz, Seville, Spain; 6 Immunology Service, Unit of Clinical Laboratories, Institute of Biomedicine of Seville, IBiS/Virgen del Rocío University Hospital/CSIC/University of Seville, Seville, Spain; 7 Internal Medicine Service, Hospital Viamed Santa Ángela de la Cruz, Seville, Spain

**Keywords:** celiac disease, duodenal biopsy, HLA genotyping, iron deficiency, seronegative celiac disease

## Abstract

**Introduction:**

Celiac disease (CD) is an immune-mediated enteropathy triggered by gluten ingestion in genetically predisposed individuals and represents a frequent but underdiagnosed cause of iron deficiency (ID). Screening for CD is recommended in unexplained ID, yet data on its prevalence among patients with absolute ID remain limited. This study aimed to determine the frequency of confirmed CD and Suspected Seronegative CD at Marsh 1 (SSCDM1) and to characterize their clinical, serological, histological, and immunogenetic profiles.

**Methods:**

A retrospective study was conducted within the ViaIron cohort including 86 individuals (≥14 years) with absolute ID (ferritin <50 ng/mL), with or without anemia, and no prior CD diagnosis. All underwent serological testing, duodenal biopsy with CD8^+^ intraepithelial lymphocyte staining, and HLA-DQ2/DQ8 genotyping. SSCDM1 was defined by Marsh 1 histology and permissive HLA genotypes in seronegative patients.

**Results:**

Among 86 participants (93% female, median age 41 years), 14% had confirmed CD (Marsh 3) and 39.5% had SSCDM1. Only one-third of confirmed cases were seropositive, and 77% were asymptomatic. SSCDM1 patients closely resembled overt CD, showing low ferritin, hepcidin, and IgA levels but normal inflammatory markers, suggesting a shared malabsorption-driven, non-inflammatory mechanism of iron depletion. Autoimmune comorbidities, particularly autoimmune thyroiditis, and permissive HLA-DQ2/DQ8 haplotypes were highly frequent in both groups.

**Discussion:**

Nearly half of participants exhibited either overt or seronegative CD, revealing a continuum between both conditions. These findings highlight a frequently under-recognized, seronegative, immune-mediated duodenal injury consistent with possible gluten-sensitive enteropathy, which may escape detection by standard serological screening.

## Graphical Abstract

**Figure:**
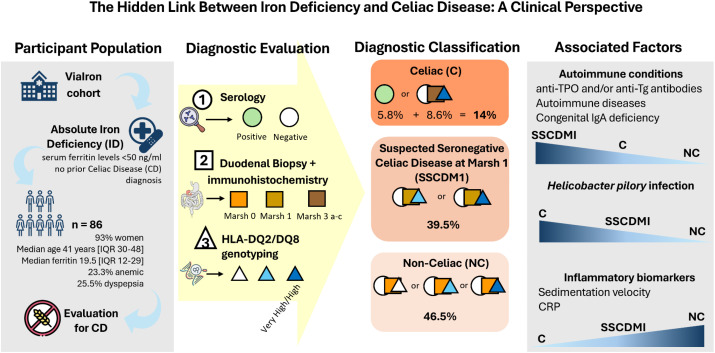

## 1 Introduction

Celiac disease (CD) is an autoimmune enteropathy triggered by gluten ingestion in genetically predisposed individuals. The ensuing response of the innate and adaptive immune system, both humoral and cellular, leads to intestinal inflammation and villous atrophy in the small intestine (1). The clinical presentation of CD is highly variable, including both gastrointestinal and extraintestinal symptoms (2). These manifestations can reflect malabsorption of iron and other micronutrients (3), or may arise from other associated conditions (4). Furthermore, CD is associated with an increased risk of complications, such as intestinal lymphoma (5). CD represents a global health-care concern (6). Its overall prevalence in the general population ranges from 0.5 to 2%. However, the disease is often underdiagnosed due to a high proportion of clinically silent cases, in which intestinal damage occurs despite the absence of symptoms (7). It is estimated that, on average, 70% of cases escape diagnosis and treatment (8).

Iron deficiency (ID) is one of the most frequent micronutrient deficiencies worldwide and a leading cause of anemia (9). However, most available data are derived from cohorts restricted to iron deficiency anemia (IDA) or based primarily on serological screening, potentially overlooking patients with absolute ID without anemia and seronegative forms of CD. Although its etiology is often linked to inadequate dietary intake, chronic blood loss, or malabsorption disorders, CD is a well-established cause of ID as iron absorption is reduced in patients with CD due to duodenal villous atrophy (the duodenum is the specific site of iron absorption) (10, 11). Recent evidence indicates that ferritin levels below 50 ng/mL identify early stages of absolute ID associated with increased iron absorption and hepcidin suppression, even in the absence of anemia (12, 13).

The current diagnosis protocol is based on the “four out of five rule”, which states that CD can be diagnosed when four of the following five criteria are met: 1) typical symptoms such as diarrhea and malabsorption; 2) positive celiac-specific antibodies; 3) presence of HLA-DQ2 and/or DQ8 haplotypes; 4) intestinal damage (e.g., villous atrophy); and 5) clinical improvement on a gluten-free diet (GFD). This rule also helps classify CD subtypes, including seronegative CD (lacking criterion 2), potential CD (lacking 4), non-classical CD (lacking 1), and non-responsive CD (lacking 5).

Although duodenal biopsy remains the gold standard for confirming or excluding CD, serological testing represents a reliable and widely used screening tool (14). In clinical practice, diagnosis is usually based on concordance between serological and histological findings; however, up to 10% of patients may lack positive antibodies (15), underscoring the importance of biopsy and HLA-DQ2/DQ8 genotyping to support diagnosis in seronegative individuals (16). The diagnosis is further reinforced when symptoms improve following a GFD (17). In diagnostically ambiguous cases, particularly seronegative patients with mild histological changes, HLA-DQ2/DQ8 genotyping provides valuable information. Although not diagnostic on its own, the absence of permissive HLA alleles effectively rules out CD, while their presence supports further evaluation.

As mentioned before, a particular diagnostic challenge is posed by seronegative subjects with preserved villous architecture and isolated intraepithelial lymphocytosis (Marsh 1). In this context, we use the term Suspected Seronegative Celiac Disease at Marsh 1 (SSCDM1) as an operational definition to describe seronegative individuals with Marsh 1 histology, permissive HLA-DQ2/DQ8 genotypes, and no alternative identified cause of duodenal lymphocytosis, investigated in the setting of absolute ID. This construct derived from previous descriptions of seronegative gluten-sensitive enteropathy, including molecular evidence of mucosal immune activation in genetically susceptible, seronegative patients with Marsh lesions, as reported by Ierardi et al. (18), but does not imply a definitive diagnosis of CD.

A strict GFD remains the only effective treatment for CD. Upon gluten withdrawal, celiac-specific antibodies progressively decline and typically normalize in parallel with mucosal recovery (19). The frequency of seronegative forms appears to increase with age, which is particularly relevant when investigating ID, as its highest incidence occurs in older adults (20).

Despite current guideline recommendations to screen for CD in cases of unexplained ID, reported prevalence estimates remain highly variable, ranging from 3% to 5% in most retrospective cohorts (21). However, reported prevalence varies widely depending on study design and diagnostic strategy, particularly with respect to inclusion criteria, ID definitions, and reliance on serological screening. Studies restricted to patients with IDA or excluding seronegative individuals are likely to underestimate seronegative or mild forms of CD, especially in patients with absolute ID without anemia.

Therefore, this study aimed to determine the frequency of confirmed celiac disease and SSCDM1 among patients with absolute ID (ferritin <50 ng/mL), to assess the diagnostic utility of a comprehensive screening strategy including duodenal biopsy and HLA genotyping in seronegative cases, and to characterize the clinical, serological, histological, and immunological profile of these patients.

## 2 Materials and methods

### 2.1 Study design and population

This retrospective and observational study was conducted within the ViaIron cohort, an ongoing single-centre clinical cohort based in an internal medicine outpatient clinic and designed to investigate the causes, diagnostic pathways, and biological correlates of absolute ID in routine clinical practice. The study included individuals aged 14 years or older who consecutively attended the outpatient clinic at Hospital Viamed Santa Ángela de la Cruz in Seville, Spain, between July, 2020, and February, 2025, and who agreed to undergo upper gastrointestinal endoscopy and genotyping analysis as part of the diagnostic work-up for ID. Participants were consecutively enrolled throughout the study period as part of routine clinical practice, without predefined sampling targets or time-based selection. All participants had absolute ID, with or without anemia, under study, without indication for blood or blood-derivative transfusion, and whose doctor requested a comprehensive diagnostic work-up to investigate the cause of the ID.

The classification of absolute ID was based on a ferritin threshold of 50 ng/mL, as recently recommended (12, 13). Exclusion criteria included pregnancy, breastfeeding, and hospitalization. The study was approved by the local Ethics Committee of the Virgen Macarena and Virgen del Rocío University Hospitals (code: 0465-N-22; CEI_03/2022) and was conducted in accordance with the Declaration of Helsinki.

### 2.2 Patient work-up

Participants underwent a diagnosis approach as follows:

1. History and clinical examination for all participants including autoimmune comorbidities, *Helicobacter pylori* (*H. pylori*) infection, and family history. Clinical expression was classified into three main categories. First, symptoms related to the inclusion criteria, namely ID with or without anemia, were common and included fatigue, hair loss, brittle nails, and exertional dyspnea, reflecting ID. Second, gastrointestinal symptoms were reported, especially dyspeptic complaints such as bloating, borborygmi, and abdominal distension, as well as diarrhea and/or constipation. It is important to note that patients were selected based on ID and/or IgA deficiency, not on digestive symptoms; gastrointestinal complaints were recorded secondarily. Third, some participants presented with manifestations related to immune-mediated or inflammatory processes, including a variety of autoimmune diseases.
2. Hematological, biochemical and immunological determinations including iron-related biomarkers for absolute ID and IgA, IgG, IgM and IgE quantification in all participants.
3. Serology markers for CD in all participants: IgA and, in cases of congenital IgA deficiency, IgG antibodies against deamidated gliadin and tissue transglutaminase.
4. Endoscopy. Patients with positive serology and patients with suspected seronegative CD were referred for duodenal biopsy. Tissue specimens were taken from the antrum, angulus, and corpus of the stomach. Four duodenal biopsies (two from the bulb and two from the second portion of the duodenum) were obtained. Endoscopy was performed to investigate the origin of ID, regardless of whether another potential cause (e.g., menorrhagia) was present.
5. HLA-DQ2/DQ8 haplotypes genotyping for potential CD cases. Genotyping was not required for the diagnostic classification of clearly celiac cases (seropositive or Marsch 3) or histologically normal cases.

### 2.3 Celiac disease diagnosis criteria

#### 2.3.1 Serology

Detection of tissue transglutaminase IgA (tTG-IgA) and deamidated gliadin peptide IgA (GliadinDP-IgA) was performed using the EliA Celikey IgA and EliA GliadinDP IgA assays (Thermo Fisher Scientific) on the Phadia 250 platform, following the manufacturer’s instructions. Briefly, assays quantify specific IgA antibodies against recombinant human tTG and deamidated gliadin peptides by fluorescence immunoassay. Results were expressed in EliA units/mL and interpreted as negative (<7), equivocal (7–10), or positive (>10), according to manufacturer’s cut-offs. Detailed technical procedures are provided in **Supplementary Material**.

#### 2.3.2 Histology

Patients with positive serology and patients with suspected seronegative CD (negative serology but persistent ID and suggestive symptoms) were referred for duodenal biopsy. Marsh classification was used for histological confirmation of CD (22). At least four duodenal biopsies were obtained from each patient, two from the duodenal bulb and two from the second portion of the duodenum. Samples were fixed in 10% buffered formalin and embedded in paraffin. Sections of 4 µm thickness were stained with hematoxylin and eosin. Histological changes were classified according to the traditional Marsh classification, as follows: Marsh 0, normal mucosa; Marsh 1, intraepithelial lymphocytes (IELs) with preserved villous architecture; Marsh 2, crypt hyperplasia with IELs; and Marsh 3, villous atrophy, subdivided into partial (3a), subtotal (3b), and total atrophy (3c).

Immunohistochemical staining for CD8 was performed on formalin-fixed, paraffin-embedded sections using a commercially available monoclonal antibody against human CD8 (Vector Laboratories, Milan, Italy), following the manufacturer’s instructions. Antigen retrieval was carried out by heat-induced epitope retrieval in citrate buffer (pH 6.0), and visualization was achieved using a peroxidase-based detection system. Positive cells were quantified by counting CD8+ IELs per 100 enterocytes in at least five well-oriented villi. Microscopic observations were performed using an Olympus BX41 light microscope.

#### 2.3.3 Genotyping and classification based on the risk attributed to genetics

For HLA-DQ2/DQ8 genotyping (23), DNA was extracted using the Chelex method, followed by PCR amplification of the polymorphic second exon of the HLA-DRB1 and DQB1 genes. Allele identification was performed by dot-blot analysis using sequence-specific oligonucleotide (SSO) probes. The risk of developing CD according to the HLA-DQ genotype was classified as very high/high, moderate, or low. According to Núñez et al. (24), genotypes including DQ2.5 were classified as high or very high risk, depending on the accompanying haplotype. Moderate risk included patients carrying at least one DQB1*03:02 (DQ8) or DQ2.2 haplotype, or a combination of both. Low risk included patients carrying one or two DQA1*05 alleles in the absence of DQ2.2, DQ8, or DQ2.5. Finally, no-risk, no permissive or negative patients were those lacking any of the aforementioned alleles or haplotypes.

#### 2.3.4 Definitions and classification of celiac disease

Patients were diagnosed as Celiac under the following conditions:1) Presence of autoantibodies, regardless of Marsh classification and genetic profile; and 2) Seronegative individuals with Marsh grade 3 classification (a, b or c) and high/very high or moderate genetic risk. Patients were defined as Suspected Seronegative Celiac Disease at Marsh 1 (SSCDM1) if they were seronegative, presented lymphocytic infiltration demonstrated by immunohistochemistry, compatible with Marsh grade 1 histology and a permissive HLA genetic profile (high/very high or moderate risk) (18). This classification was used for study purposes as a descriptive and hypothesis-generating category and does not constitute a definitive diagnosis of CD. Non-celiac individuals were defined as those with a normal duodenal biopsy, including normal histology and negative histochemical findings, regardless of their HLA genotype, and with negative autoantibodies. Marsh 0 represents the earliest stage of CD, characterized only by the presence of specific antibodies or by histological reversion from a previous Marsh stage as a consequence of a GFD.

### 2.4 Laboratory methods

Serum immunoglobulins were measured using an immunoturbidimetric method on the AU 5800 analyzer (Beckman Coulter), with specific reagents provided by Beckman Coulter. Calibrators and control materials at three concentration levels, also supplied by the manufacturer, were used to ensure accuracy and precision. IgA deficiency was defined as serum IgA levels below 10 mg/dL. Iron-related and hematological parameters, as well as inflammatory biomarkers, were determined using standardized clinical laboratory techniques, as previously described (13, 25). All analyses were performed after overnight fasting at the Laboratory Service of Hospital Viamed Santa Ángela de la Cruz. Detailed analytical procedures are provided in **Supplementary Material**. Diagnosis of *H. pylori* infection was performed using the urea breath test, gastric biopsy, or both, depending on clinical judgment and availability.

### 2.5 Statistical analysis

Given the retrospective design, the sample size was pragmatic and based on all consecutive eligible participants with complete diagnostic evaluation within the study period; therefore, no *a priori* power calculation was performed. Continuous variables were expressed as medians and interquartile ranges [IQR], and categorical variables as the number of subjects and percentage (%). Non-parametric tests were performed after the Kolmogorov–Smirnov test, which confirmed that variables did not follow a normal distribution. Group differences between categorical and continuous values were determined using Chi-square test and Mann–Whitney U-test, respectively. p-values < 0.05 were considered statistically significant. Statistical Package for Social Sciences software (SPSS 20.0) and GraphPad Prism 9.0 (GraphPad Software) were used for statistical analysis and graphs generation, respectively.

## 3 Results

### 3.1 Baseline characteristics and celiac disease diagnosis

A total of 86 participants met the inclusion criteria, most of whom were women with a median age of 41 years (range of 14 to 85 years). The 76% of participants had ferritin levels below 30 ng/mL and 23.3% were anemic. These and additional parameters are detailed in **Table 1**. At the time of evaluation, none of the participants were adhering to a GFD.

**Table 1:** Baseline characteristics of the participants.

Number of participants	n=86
Female, n (%)	80 (93)
Age (years)	41 [30-48]
BMI (Kg/m^2^)	24.3 [21.3-29]
Ferritin (ng/mL)	19.5 [12-29]
Ferritin <30 ng/mL, n (%)	65 (76)
Hb (g/dL)	13 [12.3-13.9]
Participants with anaemia, n (%)	20 (23.3)
Transferrin (mg/mL)^a^	299 [265-334]
TfSI (%)^b^	17 [9.2-28]
Sideremia (µg/dL)^b^	62.5 [41-90]
sTfR (mg/L)^c^	1.6 [1.3-2.1]
Hepcidin (ng/mL)^d^	2 [1.4-3.4]

^a,b,c,d^Data available for 83, 84, 79 and 42 participants, respectively. Anemia is defined as hemoglobin levels below 13 g/L for men and below 12 g/L for women. Percentages have been rounded per convention. BMI, body mass index; Hb, Hemoglobin; TfSI, transferrin saturation index; sTfR, soluble transferrin receptor. Continuous variables are expressed as the medians and interquartile ranges [IQR]. Categorical variables are expressed as numbers and percentages.

**Figure 1:**
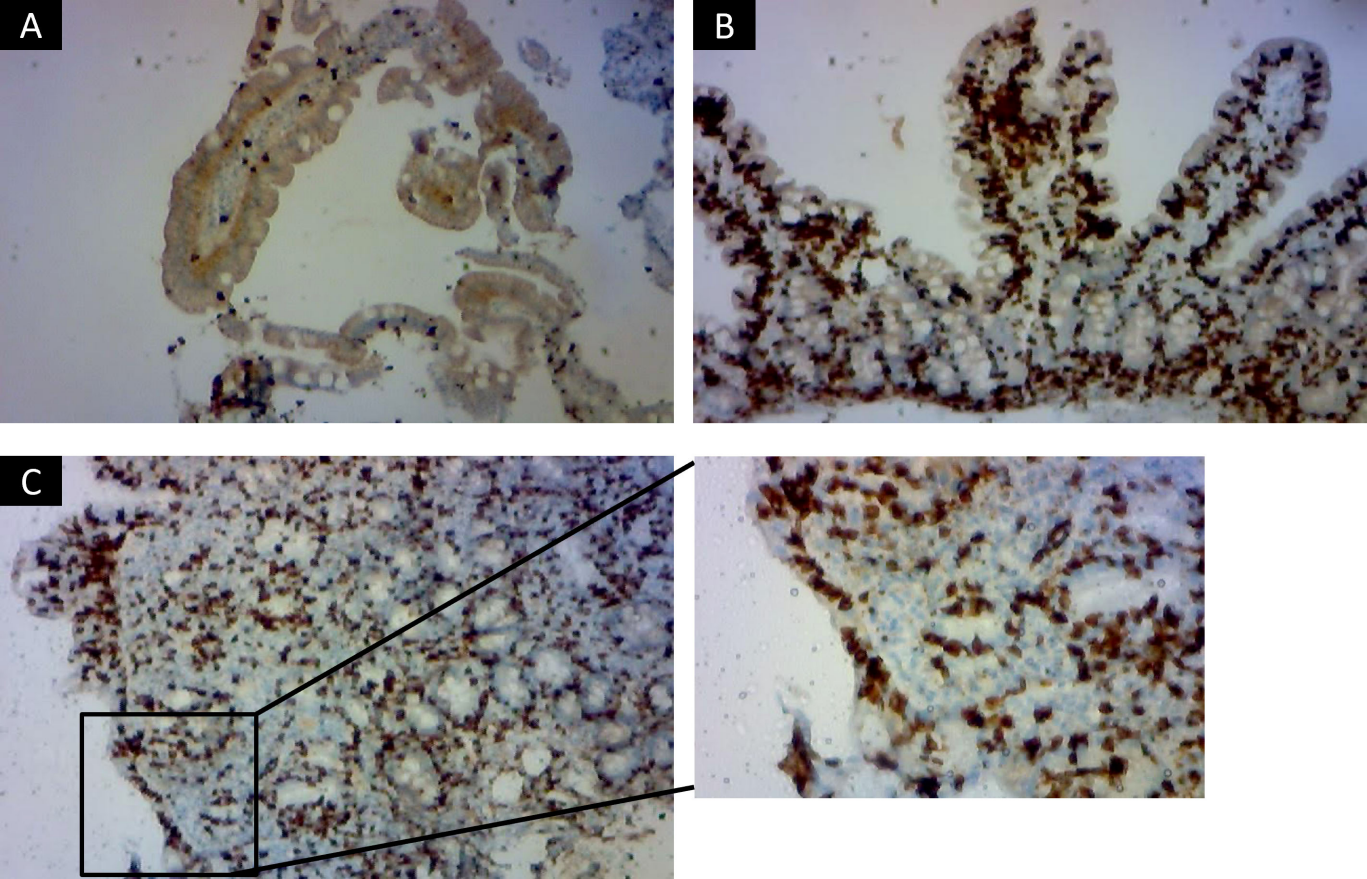
Immunohistochemical staining for CD8+ intraepithelial lymphocytes in duodenal biopsies across histological stages of celiac disease. Representative immunohistochemistry images showing CD8+ lymphocyte distribution in duodenal mucosa from three patients classified according to Marsh criteria. Marsh 0 Non-celiac subject **(A)**, with preserved villous architecture and a physiological number of intraepithelial CD8+ lymphocytes scattered along the epithelial lining, consistent with a normal histological profile (x100). Marsh 1, representing a case of Suspected Seronegative Celiac Disease at Marsh 1 **(B)**, in which the villous architecture is maintained but there is a marked increase in intraepithelial CD8+ lymphocytes, typical of genetically predisposed, seronegative individual (x400). Marsh 3 c **(C)** with confirmed celiac disease, showing total villous atrophy, crypt hyperplasia, and dense intraepithelial infiltration by CD8+ lymphocytes, characteristic of active disease (x100 and detail in x400). Images were acquired using an Olympus BX41 optical microscope.

**Figure 2:**
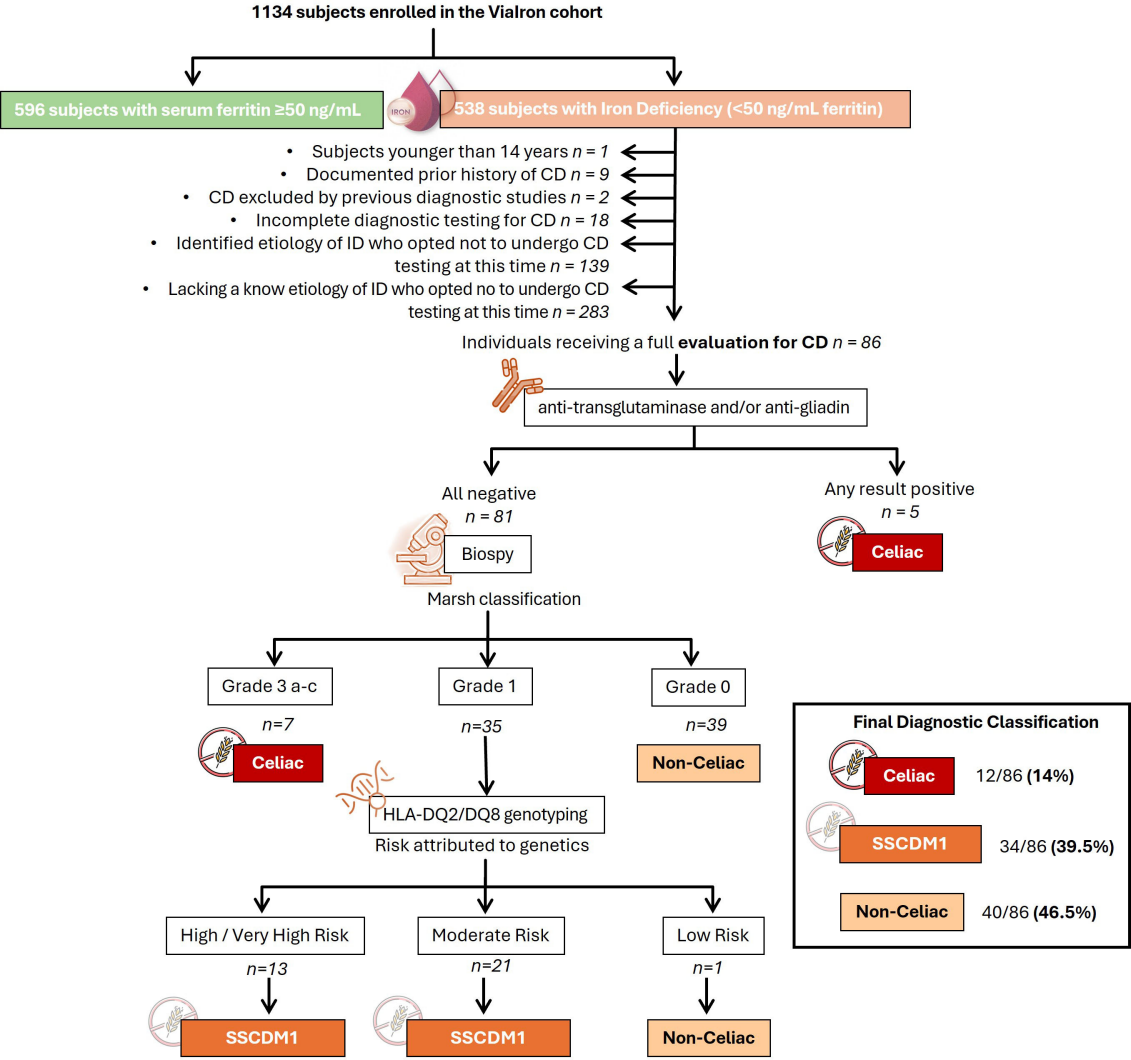
Diagnostic flowchart for suspected celiac disease in patients with absolute iron deficiency (ferritin <50 ng/mL) of unknown origin enrolled in the ViaIron cohort. The diagram illustrates the diagnostic algorithm applied to 1,134 individuals enrolled in the ViaIron cohort, of whom 538 presented with absolute iron deficiency (ferritin <50 ng/mL). The following were excluded from the analysis: individuals under 14 years of age, those with a previously confirmed or ruled-out diagnosis of celiac disease in another center, those with incomplete diagnostic workup, and those with a known or unknown non-celiac cause of iron deficiency who declined further celiac disease evaluation. Known causes of iron deficiency included bariatric surgery or intragastric balloon placement, malnutrition (including eating disorders), menorrhagia (associated with myoma, endometriosis, or bleeding disorders), coagulopathy linked to recurrent miscarriages, chronic blood loss (e.g., from hemorrhoids, epistaxis, or gastrointestinal neoplasia), prolonged use of proton pump inhibitors, and frequent blood donation. The diagnostic process included serologic testing (anti-transglutaminase and anti-gliadin antibodies) followed by duodenal biopsy in seronegative cases. Histological classification was performed according to the Marsh criteria. Marsh 0 represents the earliest stage of celiac disease, characterized only by the presence of specific antibodies or by histological reversion from a previous Marsh stage as a consequence of a gluten-free diet. In patients with suspected Marsh 1 lesions, HLA-DQ2/DQ8 genotyping was used to assess genetic susceptibility and define cases as Suspected Seronegative Celiac Disease at Marsh 1. The number of subjects at each stage of the participant selection and diagnostic pathway is indicated. Declined further evaluation refers to patients in whom additional diagnostic procedures (e.g., upper endoscopy and/or HLA genotyping) were offered as part of routine clinical care but were not performed due to patient preference. CD, Celiac Disease; SSCDM1, Suspected Seronegative Celiac Disease at Marsh 1.

Immunohistochemical staining for CD8 was used to support the histological evaluation of duodenal biopsies, allowing for detailed assessment of IELs infiltration across the spectrum of mucosal lesions. Representative examples from patients are shown in **Figure 1**, highlighting the progressive increase in CD8+ IELs from normal mucosa to severe villous atrophy. The diagnostic algorithm was applied to all participants until they were classified into one of three diagnostic categories: celiac disease, SSCDM1, or Non-celiac. As shown in **Figure 2**, of the 1,134 individuals initially enrolled in the ViaIron cohort, 538 (47.4%) fulfilled criteria for absolute ID (ferritin levels <50 ng/mL). Among them, a substantial proportion did not undergo CD evaluation due to predefined exclusion criteria or patient decision, including age <14 years (n=1), previous diagnosis or exclusion of CD (n=11), incomplete diagnostic work-up (n=18), or the presence of an identified (n=139) or unidentified (n=283) alternative cause of ID with refusal of further CD testing based on patient preference. Consequently, 86 individuals (16% of those with absolute ID) underwent a complete diagnostic evaluation for CD. Five (5.8%) tested positive for antibodies and were directly diagnosed as Celiac. Among seronegative individuals, 7 (8.6%) presented with Marsh grade 3 a-c lesions and were also classified as Celiac. Forty participants (46.5%) were classified as Non-celiac, based on Marsh 0 histology (39 subjects, 45.3%) or Marsh 1 lesions along with non-permissive haplotypes (one subject, 1.2%) despite presenting symptoms and absolute ID. SSCDM1 was identified in 34 participants (39.5%), all of whom were seronegative, showed Marsh grade 1 lesions, and carried a high/very high (n = 13) or moderate (n = 21) genetic risk. In summary, within this highly selected subgroup, the final diagnostic classification included 12 patients (14%) as Celiac, 34 (39.5%) as SSCDM1, and 40 (46.5%) as Non-celiac. When considering only anemic participants, the distribution was as follows: 3 patients (15%) were classified as Celiac, 8 (40%) as SSCDM1, and 9 (45%) as Non-celiac.

### 3.2 Clinical symptoms, autoimmune comorbidities, *Helicobacter pylori* infection, family history, and genotype distribution by celiac status

Clinical symptoms at presentation are summarized in **Supplementary Table 1**. Overall, a substantial proportion of patients were asymptomatic, particularly in the non-celiac group (52.5%). Across all categories, the most frequently reported manifestations were symptoms associated with absolute ID, including fatigue/adinamia, hair loss, brittle nails, and angular cheilitis. At least one ID–related symptom was present in 41.7% of patients classified as Celiacs, 52.9% of patients with SSCDM1, and 25% of non-celiac patients. Fatigue/adinamia was the most common symptom, reported by 33.3%, 44.1%, and 22.5% of patients in the Celiac, SSCDM1, and non-celiac groups, respectively.

Dyspeptic gastrointestinal symptoms were reported by a minority of patients in all groups. At least one dyspeptic symptom was present in 16.7% of patients with CD, 11.8% of patients with SSCDM1, and 20% of non-celiac individuals, being postprandial bloating the most frequently reported dyspeptic manifestation. Non-dyspeptic gastrointestinal symptoms, including diarrhea and constipation, were uncommon across all diagnostic categories.

A detailed assessment of comorbid conditions, with a particular focus on autoimmune diseases, was conducted across the three status groups (**Table 2**). This analysis aimed to explore the potential association between celiac disease status and the presence of immune-mediated disorders. Autoimmune thyroiditis, defined by the presence of anti-thyroid peroxidase antibodies (anti-TPO) and/or anti-thyroglobulin antibodies (anti-Tg), was the most frequent immune-related condition observed in the cohort. It was present in 50% (17/34) of participants with SSCDM1, compared to 17% (2/12) in those with confirmed Celiac disease and 25% (10/40) in Non-celiac group. Other autoimmune conditions, including autoimmune hepatitis, Raynaud’s phenomenon, and psoriasis, were observed only in the SSCDM1 group, with a frequency ranging from 3% to 6%. Notably, the highest frequency of coexisting autoimmune conditions was observed in the SSCDM1 group, where 8% (4/34) of individuals presented with two or more autoimmune diseases. Congenital IgA deficiency was also more frequently observed in the SSCDM1 group compared to others.

**Table 2:** Frequency and types of antoimmune diseases according to celiac disease status.

Associated conditions		Celiac (Marsh 3)	SSCDM1	Non-celiac
Autoimmune condition	Autoimmune thyroiditis anti-TPO and/or anti-Tg	2/12 (17)	17/34 (50)	10/40 (25)
	Autoimmune hepatitis	1/12 (8.3)	1/34 (3)	0/40 (0)
	Raynaud’s phenomenon	0/12 (0)	2/34 (6)	0/40 (0)
	Sarcoidosis	0/12 (0)	1/34 (3)	0/40 (0)
	Psoriasis	0/12 (0)	1/34 (3)	0/40 (0)
	Antiphospholipid syndrome	0/12 (0)	1/34 (3)	0/40 (0)
	Alopecia areata	0/12 (0)	1/34 (3)	2/40 (5)
	Inflammatory proctitis	0/12 (0)	1/34 (3)	0/40 (0)
Subjects with at least one autoimmune condition		3/12 (25)	21/34 (62)	11/40 (27.5)
Subjects with ≥2 autoimmune conditions		0/12 (0)	4/34 (8)	1/40 (2.5)
Congenital IgA deficiency (<10 mg/dL)		1/12 (8.3)	4/33 (12)	1/40 (2.5)
Migraine		1/12 (8.3)	1/34 (3)	0/40 (0)

Autoimmune thyroiditis was diagnosed based on positivity for anti-thyroid peroxidase (anti-TPO) and/or anti-thyroglobulin (anti-Tg) antibodies. Percentages have been rounded per convention. Variables are expressed as numbers and percentages. SSCDM1, Suspected Seronegative Celiac Disease at Marsh 1.

**Figure 3:**
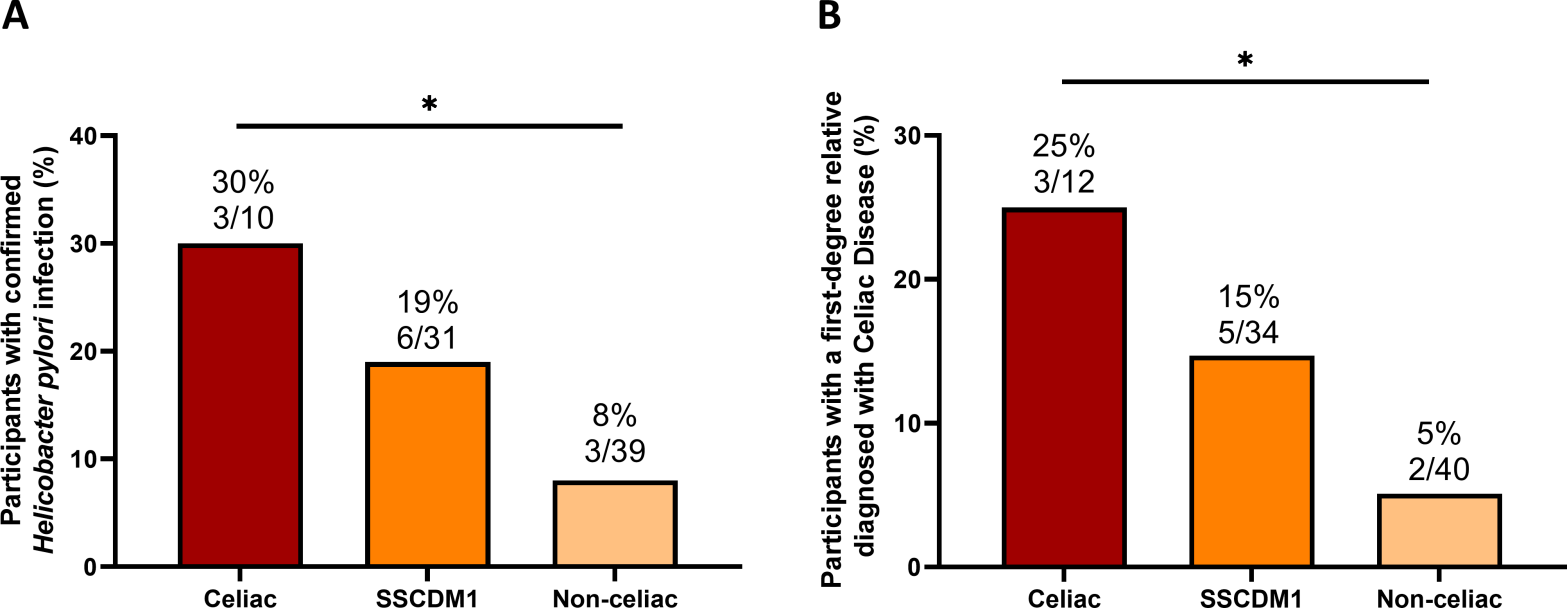
Frequency of *Helicobacter pylori* infection and family history of confirmed celiac disease diagnosis across celiac disease status. Frequency of *Helicobacter pylori* infection among participants for whom testing data were available **(A)** and frequency of positive family history of celiac disease in first-degree relatives, defined as parents or siblings **(B)**. Analyses were restricted to participants with available data for each variable; therefore, the number of subjects per group differs from the total cohort. SSCDM1, Suspected Seronegative Celiac Disease at Marsh 1. Group comparisons were performed using the Chi-square test. Only comparisons with *p*-value < 0.1 are indicated. *0.01≤p-value<0.05.

Among participants for whom *H. pylori* testing was available, the frequency of infection was 30% (3/10) in the confirmed Celiac group, 19% (6/31) in the SSCDM1 group, and 8% (3/39) in the Non-celiac group (**Figure 3A**). A positive family history of CD in first-degree relatives (defined as parents, siblings or children) was identified in 25% (3/12) of participants with confirmed Celiac, 15% (5/34) of those classified as SSCDM1, and 5% (2/40) of non-celiac individuals (**Figure 3B**).

Analysis of the distribution of HLA-DQ2/DQ8 genotypes revealed a strong association between genetic risk and celiac disease status (**Table 3**). Among confirmed Celiac patients, 75% (6/8) carried a high/very high genotype, while the remaining 25% (2/8) were classified as moderate to low risk. Importantly, none of the confirmed CD cases exhibited non-permissive genotypes. In contrast, nearly half of the non-celiac group (44.5%, 12/27) lacked permissive HLA alleles, with only 18.5% (5/27) carrying high/very high-risk genotypes and 37% (10/27) in the moderate/low-risk category.

**Table 3:** Distribution of HLA-DR and DQ risk haplotypes according to celiac disease status.

Risk of developing CD based on HLA-DQ2/DQ8 genotype	Celiac (Marsh 3)	SSCDM1	Non-celiac
Very high/High	6/8 (75)	13/34 (38)	5/27 (18.5)
Moderate/Low	2/8 (25)	17/34 (50)	10/27 (37)
No permissive/Negative	0/8 (0)	0/34 (0)	12/27 (44.5)

Percentages have been rounded per convention. SSCDM1, Suspected Seronegative Celiac Disease at Marsh 1. Variables are expressed as numbers and percentages.

### 3.3 Immunoglobulin profile and inflammatory soluble biomarkers across celiac diagnostic categories

**Figure 4:**
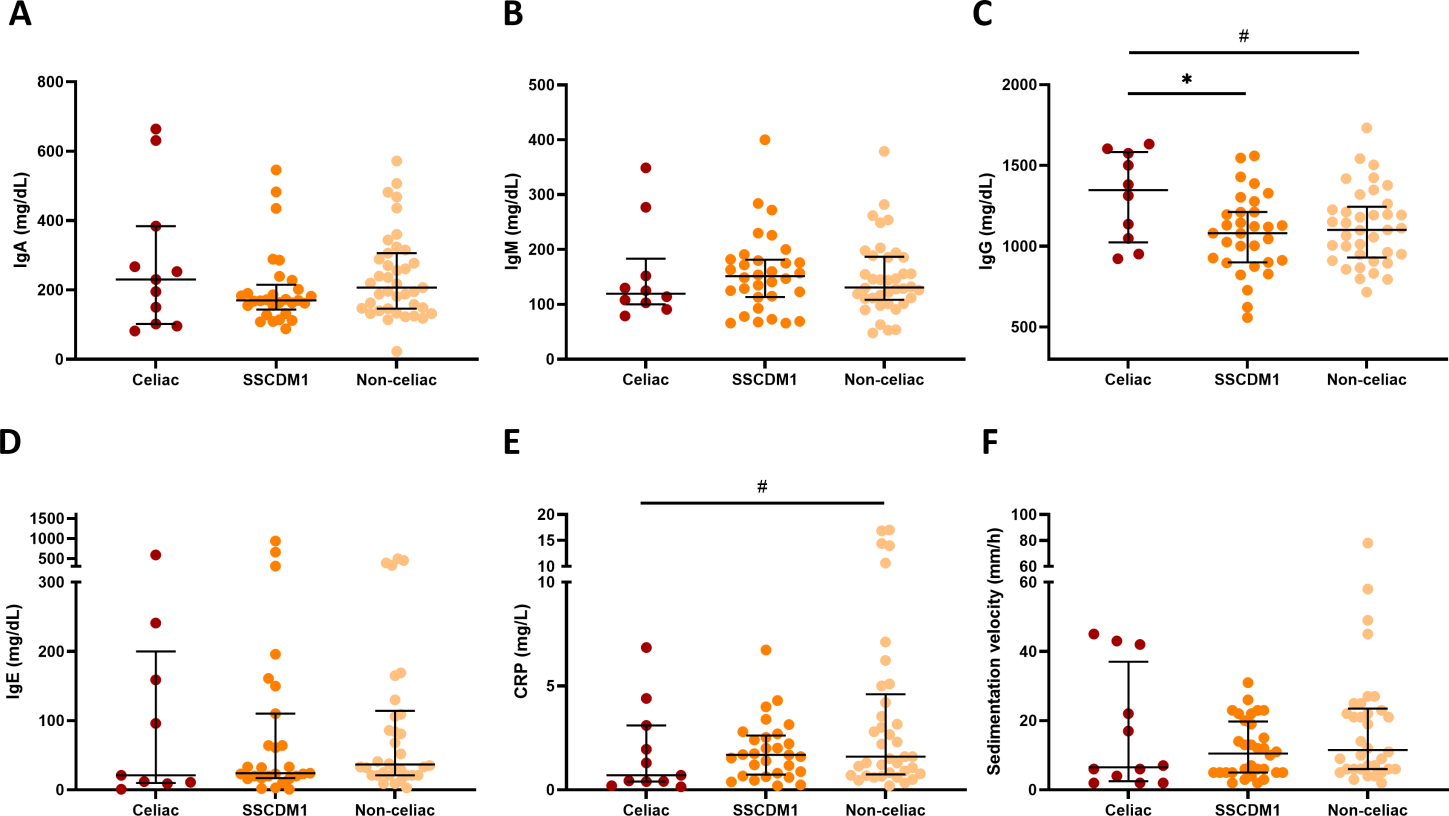
Serum immunoglobulin concentrations and inflammatory biomarkers in relation to celiac disease status. Total IgA **(A)**, IgM **(B)**, IgG **(C)**, IgE concentrations **(D)**, serum C-reactive protein **(E)** and sedimentation velocity **(F)** in participants classified as Celiac, Suspected Seronegative Celiac Disease at Marsh 1 and Non-Celiac. Individuals with congenital IgA deficiency (<10 mg/dL) were excluded from the IgA analysis to avoid distortion of group comparisons. SSCDM1, Suspected Seronegative Celiac Disease at Marsh 1; CRP, C-reactive protein. Differences between categories were determined using Mann–Whitney U-test. Only comparisons with *p*-value < 0.1 are indicated. # 0.1>p-value≥0.05; *0.01≤p-value<0.05.

Total serum immunoglobulin concentrations (IgA, IgM, IgG, and IgE) were compared between participants classified as Celiac, SSCDM1 and Non-celiac disease (**Figures 4A-D**). Individuals with congenital IgA deficiency (<10 mg/dL) were excluded from the IgA analysis to avoid distortion of group comparisons. After this exclusion, no statistically significant differences in IgA levels were observed among the groups, although values tended to be lower in the SSCDM1 group. IgG levels in the Celiac group remained significantly higher compared to SSCDM1 and Non-celiac participants (p < 0.05). IgM and IgE levels did not differ significantly, although a general upward trend was observed in SSCDM1 and Non-celiac participants compared to Celiac group. **Figures 4E, F** shows inflammatory biomarkers stratified by diagnostic group. Despite no statistical significance, CRP and sedimentation velocity demonstrated a trend toward being decreased in the Celiac and SSCDM1 group. For IL-6 and TNF-α, the number of available data points was very limited across the cohort (n = 20 in both cases), and therefore, comparative analysis between groups does not allow for any meaningful conclusions to be drawn.

### 3.4 Iron-related biomarkers and hematological indices based on celiac disease status

**Figure 5:**
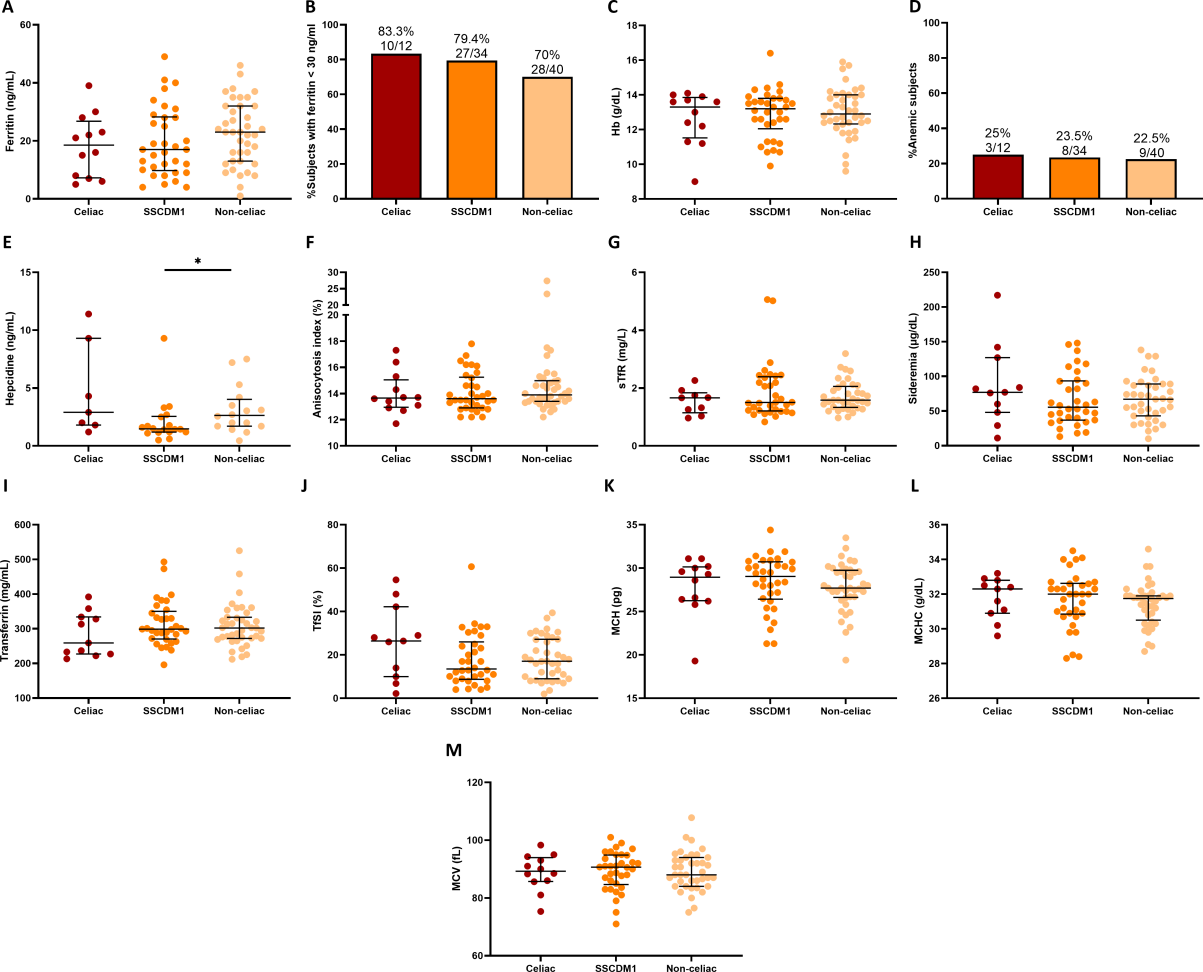
Iron-related metabolism and hematological markers by celiac disease status. Serum ferritin **(A)**, percentage of subjects with ferritin levels <30 ng/mL **(B)**, Hb **(C)**, percentage of subjects with anemia **(D)**, hepcidin **(E)**, anisocytosis index **(F)**, sTfR **(G)**, sideremia **(H)**, transferrin **(I)**, TfSI **(J)**, MCH **(K)**, MCHC **(L)** and MCV **(M)** in Celiac, Suspected Seronegative Celiac Disease at Marsh I and Non-Celiac participants. Anemia was defined as hemoglobin <13 g/dL in men and <12 g/dL in women. SSCDM1, Suspected Seronegative Celiac Disease at Marsh 1; Hb, hemoglobin; sTfR, soluble transferrin receptor; TfSI, transferrin saturation index; MCH, mean corpuscular hemoglobin; MCHC, mean corpuscular hemoglobin concentration; MCV, mean corpuscular volume. Differences between categories were determined using Mann–Whitney U-test. Only comparisons with *p*-value < 0.1 are indicated. *0.01≤p-value<0.05.

As illustrated in **Figure 5**, iron-related and hematological biomarkers were analyzed according to celiac disease status. Overall, no statistically significant differences were detected among groups; however, some trends were observed. Ferritin concentrations tended to be lower in Celiac and SSCDM1 participants compared to Non-celiac individuals and the proportion of individuals with ferritin <30 ng/mL was also higher in the Celiac and SSCDM1 groups (83.3%, 79.4% and 70% in the Celiac, SSCDM1 and Non-celiac participants), suggesting a more pronounced degree of iron depletion in Celiac and SSCDM1 groups despite comparable Hb value. In parallel, Celiac patients showed slightly lower transferrin levels but a higher TfSI, also compatible with a more accentuated absolute ID pattern. Given the smaller sample size of the Celiac group, it is possible that some group differences did not reach statistical significance, representing a limitation of the present analysis.

## 4 Discussion

The study provides insights into the diagnostic profile of CD among patients with absolute ID, with or without anemia. We identified a similar proportion of confirmed or suspected CD, many without gastrointestinal symptoms or positive serology, but with autoimmune diseases, low IgA, seronegative profile, and ID evidence of malabsorption rather than inflammation. These findings describe a frequently underdiagnosed, seronegative, immune-mediated duodenal alteration consistent with possible gluten-sensitive enteropathy.

Previous studies reported 5–15% prevalence of CD in IDA (20, 21, 26, 27), a range that is consistent with the 15% observed in our study. However, data were generally limited to anemic subjects and often excluded seronegatives, hindering SSCDM1 detection. We, among others, showed that anemia is not a reliable surrogate marker of iron depletion as absolute ID can occur even with normal Hb levels (13, 25). Data on absolute ID without anemia remain scarce, with small cohorts, heterogeneous ID definitions (e.g., ferritin <12 ng/mL or combined with folate deficiency) (28, 29) and with exclusion of individuals with negative celiac serology limiting the detection of the clinical entity SSCDM1 (e.g., Cosnes et al., 2013). By contrast, we used a comprehensive broader diagnostic approach with more sensitive diagnostic criteria on patients with no prior CD using a high ferritin threshold to identify early-stage absolute ID (ferritin <50 ng/mL) (12, 13), and assessing more diagnostic pillars of CD (duodenal biopsy and HLA genotyping in seronegative subjects), which may explain the high frequency of CD and SSCDM1 observed. Most population-based studies reported a 3–5% frequency of CD in unexplained IDA (21) but, our findings suggest that this may be an underestimation in cases with true iron depletion independently of anemia. Based on the ViaIron cohort, we previously reported that 11.5% of subjects with absolute ID had CD as the main underlying cause (25). Combined with present findings, this percentage may be higher than previously estimated. It is worth noting that, in routine clinical practice, oral iron therapy is usually prescribed only when ID is accompanied by anemia, and upper gastrointestinal endoscopy with stepwise duodenal biopsies is often reserved for those who fail to respond to oral supplementation. As highlighted by Iriarte-Gahete et al. (30), this approach may contribute to underdiagnosis of seronegative or mild CD forms. In our study, duodenal biopsy was performed in all cases of absolute ID regardless of anemia and even in the presence of an alternative plausible cause. This strategy, supported by HLA genotyping, likely explains the high frequency of SSCDM1 found in our study.

The concept of SSCDM1 refers to individuals with duodenal biopsy abnormalities despite negative serology (7), a scenario that poses a well-recognized diagnostic and therapeutic challenge in clinical practice. Distinguishing seronegative CD from other causes of mild duodenal injury requires a careful differential diagnosis, and uncertainty remains regarding optimal management, including the indication for a GFD (31). To address this diagnostic ambiguity, we explored permissive HLA-DQ2/DQ8 genotypes as an additional discriminative tool. In accordance with current recommendations (24), seronegative patients with Marsh 1 histology and permissive HLA haplotypes were classified as SSCDM1, whereas the absence of permissive alleles effectively ruled out CD in nearly half of non-celiac individuals.

While HLA testing alone is not diagnostic, its integration into a comprehensive diagnostic approach improves clinical decision-making and helps avoid unnecessary GFD in genetically non-susceptible individuals. Importantly, whether SSCDM1 represents an early stage of CD or a nonspecific inflammatory finding remains a matter of debate. Nevertheless, the convergence of persistent absolute ID, increased IELs, permissive HLA-DQ2/DQ8 genotypes, and a high frequency of autoimmune comorbidities supports the interpretation of SSCDM1 as a celiac-consistent enteropathy within the broader spectrum of seronegative enteropathies, rather than as an incidental histological finding.

Most striking was the coexistence of two or more autoimmune disorders in 8% of SSCDM1. This aligns with Conrad et al., that showed that autoimmune diseases frequently co-occur, among endocrine and connective tissue disorders, suggesting shared pathogenic mechanisms, genetic predispositions, or molecular mimicry (32). Furthermore, early diagnosis and adherence to a GFD may reduce the incidence of subsequent autoimmune diseases, highlighting the importance of early recognition and intervention (33). The higher frequency of autoimmune and immune-mediated conditions in SSCDM1 group, particularly autoimmune thyroiditis and multiple autoimmune disorders, suggests that these patients may indeed belong to the spectrum of true CD rather than representing nonspecific duodenal changes. However, this finding should be interpreted with caution, as the lower number of patients in the CD group may have limited our ability to detect similar associations. This clustering of comorbidities provides indirect evidence of underlying disease and may reflect a broader systemic immune dysregulation, as proposed by Ludvigsson et al., who emphasized the interplay between chronic inflammation, nutritional deficiencies, and multisystemic involvement in CD (4). In line with data describing the coexistence of autoimmune diseases in seronegative or mild histological forms of CD, our findings reinforce that SSCDM1 should not be underestimated as a benign or incidental entity. Instead, the presence of autoimmune comorbidities may serve as a clinical clue to suspect underlying CD in patients with ID and negative serology, supporting the integration of genetic and histological data to avoid misclassification. The frequent coexistence of antithyroid antibodies may also be influenced by micronutrient deficiencies, particularly iron, which plays a crucial role in thyroid function and is commonly depleted in both CD and autoimmune thyroiditis (34). Taken together, these data support a comprehensive immunological assessment and genetic testing in ambiguous cases, even in the absence of enteropathy. Long-term follow-up studies in rigorously defined seronegative CD have demonstrated that a substantial proportion of patients experience both symptomatic improvement and histological normalization after adherence to a GFD, even in the absence of positive serology or advanced villous atrophy (35). However, these findings cannot be directly extrapolated to SSCDM1 patients in the present study, as treatment response was not prospectively assessed.

In the present study, the clinical presentation was characterized by a predominance of non-specific, ID–related symptoms rather than gastrointestinal manifestations. Notably, dyspeptic symptoms were reported by a minority of patients across all diagnostic categories and showed largely overlapping distributions between celiac, SSCDM1, and non-celiac individuals. In contrast, symptoms such as fatigue, hair loss, and brittle nails were substantially more frequent, underscoring that patient selection was driven by objective ID rather than gastrointestinal symptomatology. These findings are clinically relevant, as the overall symptom profile observed in the fully evaluated cohort suggests that the study population was not enriched for gastrointestinal complaints.

In contrast to previous data, *H. pylori* infection was more frequently detected among participants classified as Celiac or SSCDM1 than among Non-celiac individuals. However, these observations should be interpreted with caution, as *H. pylori* testing was available only in a subset of participants and absolute numbers were small. Yue et al. (36) reported *H. pylori* colonization and CD association, although without evidence of a causal relationship, suggesting that *H. pylori* may indirectly influence CD development. This discrepancy may be partially explained by the ID present in all participants, a condition closely linked to *H. pylori* infection. Indeed, *H. pylori* is identified as a frequent and potentially causal factor in cases of iron-refractory or iron-dependent anemia (37), through several mechanisms, which increases gastric pH and impairs Fe³^+^ reduction to the absorbable Fe²^+^ form. Thus, the increase frequency found in our CD group may reflect a shared nutritional background rather than a direct pathogenic link. Importantly, that association does not imply causality. One possibility is that CD, through altered mucosal immunity or epithelial integrity, facilitates *H. pylori* colonization. Alternatively, *H. pylori* might act as a trigger for CD in genetically predisposed individuals. Indeed, various microorganisms, particularly viruses (recently SARS-CoV-2), are proposed as environmental factors that could increase intestinal permeability, promoting gliadin peptide exposure to antigen-presenting cells and triggering or worsening autoimmune responses in CD (38). Accordingly, most reviews recommend eradicating *H. pylori* before attributing duodenal lesions to CD, highlighting the interplay between *H. pylori*, mucosal injury, and intestinal autoimmunity, and the importance of excluding bacterial infection as a potential confounder in CD diagnosis.

ID in CD has a multifactorial origin (1, 39). Factors include duodenal villi blunting which impairs absorption in the primary site of iron uptake (39, 40), occult gastrointestinal bleeding that may further contribute to the risk of IDA (40) and, inflammation-driven mechanisms as pro-inflammatory cytokines that can stimulate hepcidin production, which inhibits iron release from enterocytes and macrophages, exacerbating ID (39, 41). In our study, patients diagnosed with CD (either confirmed or SSCDM1) showed a distinct profile in terms of inflammation. Although CD is often associated with chronic inflammation, we observed low levels of CRP and sedimentation velocity, supporting the idea that intestinal damage in CD can occur without triggering a systemic inflammatory response (15). This likely reflects the asymptomatic nature of the majority of patients in our cohort, who were diagnosed following evaluation for unexplained ID.

After excluding subjects with congenital IgA deficiency, no significant differences in IgA levels were found among diagnostic groups, although values tended to be lower in Celiac or SSCDM1 groups. Conversely, IgG levels were significantly higher in celiac patients, consistent with a systemic humoral activation and autoimmune nature of the disease. The lack of elevation in IgE further supports the absence of IgE-mediated gluten hypersensitivity, indicating that the immune response is primarily autoimmune rather than allergic. Together, these findings suggest that while CD could locally decrease IgA production, systemic IgG responses are preserved or even enhanced. This imbalance could explain the frequent seronegativity observed in IgA-deficient or IgA-low individuals, as standard CD serology predominantly targets IgA isotypes. Consequently, partial IgA deficiency may contribute to the underdiagnosis of CD in patients with ID when serological testing alone is used (42).

Regarding iron metabolism, no statistically significant differences were detected across diagnostic groups; however, consistent trends emerged that are compatible with the pathophysiological pattern of ID in CD. Ferritin levels tended to be lower in both Celiac and SSCDM1 participants compared to Non-celiac individuals, indicating a more pronounced degree of iron depletion. In line with this, the proportion of individuals with ferritin levels <30 ng/mL was higher among Celiac and SSCDM1 participants than in Non-celiac individuals. In addition, Celiac patients showed slightly lower transferrin concentrations but a higher TfSI, together suggesting a reduced iron storage pool and altered iron mobilization.

The similar frequency of anemia among groups (approximately 22–25%) further supports the notion that Hb alone is not a sensitive marker of ID. This finding reinforces the concept that the absence of anemia does not exclude clinically relevant iron depletion, which in turn may delay recognition of underlying causes such as CD. The stringent inclusion criteria (ferritin < 50 ng/mL for all participants, with most values < 30 ng/mL) likely contributed to the lack of significant intergroup differences by producing a homogeneous cohort with uniformly low iron indices.

Collectively, these results highlight the need for comprehensive iron studies in patients with unexplained ID, even in the absence of anemia or overt gastrointestinal symptoms, to avoid underdiagnosis of CD and its potential complications. Although novel immunological tools such as flow cytometric analysis of IELs subsets, detection of mucosal IgA deposits against tissue transglutaminase, or *in vitro* IL-2 responses following gluten exposure have been proposed to refine the diagnosis of seronegative CD (43–45), these techniques are not yet applicable in routine clinical practice. A pragmatic approach remains to reassess histological findings after 6–12 months of a GFD.

### 4.1 Strengths and limitations of the study

Proximal small intestinal mucosa histology is still the diagnostic gold standard and must always be performed. Considering the broad spectrum of possible lesions in CD, the Marsh classification remains valid under optimal clinical conditions, but the considerable number of diagnostic categories involved makes it prone to low inter- and intra-observer agreement. False-positive and false-negative results may arise from patchy mucosal damage, observer variability, low-grade histopathological abnormalities, or technical limitations. In this regard, stepped duodenal sampling, at least two biopsies from the bulb and two from the second portion of the duodenum, is strongly recommended to reduce sampling error and increase diagnostic accuracy. Hence, reliance on standard histological findings alone may result in failure to diagnose CD (46). Individuals with isolated IELs (Marsh 1) who are not clinically suspected of having CD may later develop the disease during follow-up (47). Although mucosal changes in CD are thought to develop gradually, whether minor lesions in asymptomatic patients represent early-stage CD remains unclear (48).

Genotyping was systematically performed in all participants classified as SSCDM1 and in those with histological findings suggestive of CD, it was not available for all non-celiac individuals. In these latter cases, genotyping was not essential for diagnostic classification, as their serology and histology were conclusive. Nevertheless, this limitation is unlikely to have affected the overall diagnostic accuracy, as the genotyping strategy was applied systematically in cases where it was clinically relevant.

Given the cross-sectional and retrospective nature of the study, all associations should be interpreted as descriptive and hypothesis-generating rather than causal. The referral-based design inevitably introduces selection bias; therefore, the proportion of confirmed CD and SSCDM1 observed should not be interpreted as population prevalence, but rather as the diagnostic yield of a systematic work-up applied to a highly selected subgroup of individuals with ID. SSCDM1 represents a heterogeneous and incompletely characterized entity. Large prospective cohorts of seronegative villous atrophy and Marsh 1 lesions have shown that only a minority of cases are ultimately attributable to CD, with a substantial proportion being idiopathic or resolving spontaneously on a gluten-containing diet. As our study did not include prospective GFD intervention or follow-up histology, gluten dependence and therapeutic response cannot be established. Accordingly, SSCDM1 should be interpreted as a descriptive, hypothesis-generating category rather than as evidence of missed or clinically silent CD.

An additional limitation relates to the broad differential diagnosis of seronegative duodenal injury. Isolated IELs and mild duodenal architectural changes may occur in a wide range of infectious, inflammatory, immune-mediated, iatrogenic, and lymphoproliferative conditions (49). In this context, concomitant *H. pylori* infection may have contributed to duodenal lymphocytosis in a subset of SSCDM1 patients. Although major alternative causes were systematically evaluated according to routine clinical practice, residual confounding by unrecognized or subclinical conditions cannot be fully excluded.

Generalizability is limited by the single-centre design and the predominance of females. Importantly, this female-skewed distribution reflects the epidemiology of absolute ID and referral patterns inherent to the internal medicine outpatient setting, rather than true sex-specific differences in CD prevalence, which cannot be inferred from the present study.

At the same time, the ViaIron cohort, conceived to investigate ID in routine clinical practice, has previously enabled detailed characterization of iron metabolism across different populations (13, 50), and provided a robust and standardized framework, including uniform serological testing, stepped duodenal biopsy, and HLA genotyping under consistent laboratory conditions, which strengthens the reliability of the histopathological and clinical findings reported herein.

As conclusion, our findings highlight a profile of seronegative, celiac-consistent enteropathy associated with absolute ID, which may escape detection by standard serological screening strategies. These insights underscore the importance of considering CD in any case of unexplained ID, with or without anemia, regardless of symptom presentation or the presence of an alternative cause of ID.

## Data Availability

The raw data supporting the conclusions of this article will be made available by the authors, without undue reservation.
