# Supplementary material for: The hidden link between iron deficiency and celiac disease: a clinical perspective
Source: Front Immunol. 2026 Feb 19;17:1744005. doi: 10.3389/fimmu.2026.1744005 (PMC12960523; doi:10.3389/fimmu.2026.1744005)
Supplement: Supplementary file 1 [file DataSheet1.docx]

**The Hidden Link Between Iron Deficiency and Celiac Disease: A Clinical Perspective**

**Supplementary Material**

**Supplementary Methods - Serological assays for celiac disease**

For tTG-IgA detection, wells were precoated with recombinant human tTG. After adding the serum and washing, any bound tTG-IgA was identified using beta-galactosidase-conjugated anti-IgA secondary antibodies. The fluorescent substrate 4-methylumbelliferyl-β-D-galactoside was then added. Once the reaction was stopped with sodium carbonate, fluorescence emission was measured in response units (RU), which were automatically generated by the software from a six-point calibration curve.

The EliA GliadinDP IgA wells are coated with synthetic deamidated gliadin peptides. If antibodies to these peptides are present in the patient specimen, they bind to their specific antigen. After washing away unbound antibodies, enzyme-labeled anti-human IgA antibodies (EliA IgA Conjugate) are added to form an antibody–conjugate complex. Following incubation and further washing, the complex is developed with a development solution. Fluorescence in the final mixture is then measured. The intensity of the fluorescence is proportional to the amount of specific IgA present in the sample. The test result is evaluated by comparing the response of the patient sample to that of the calibrators. The Phadia 250 system measures specific IgA concentrations in micrograms per liter, which are automatically converted to EliA units per milliliter using a conversion factor provided by the lot-specific code of the EliA Celikey IgA wells.

Interpretation of results was as follows: values below 7 EliA units per milliliter were considered negative, values between 7 and 10 were considered uncertain, and values above 10 were considered positive.

**Supplementary Methods – Laboratory assays**

Total serum iron (sideremia), transferrin, and soluble transferrin receptor (sTfR) were measured by photometry, and ferritin by particle-enhanced immunoturbidimetric assay on a Hitachi Cobas C702 modular analyzer (Roche Diagnostics, Rotkreuz, Switzerland).

The transferrin saturation index (TfSI) was calculated as:

TfSI (%) = [serum iron (µg/dL) × 100] / [transferrin (mg/dL) × 1.27].

Plasma hepcidin was determined by ELISA (Hepcidin25 HS ELISA, DRG Instruments), C-reactive protein (CRP) by photometry (Cobas 701, Roche Diagnostics, Mannheim, Germany), and interleukin-6 (IL-6) and tumor necrosis factor-alpha (TNF-α) using Immulite 2000 and Immulite One systems (Siemens Healthcare Diagnostics), respectively, following manufacturer instructions.

**Supplementary Table 1. Clinical symptoms distribution according to celiac disease status.**

|  | | Celiac (Marsh 3) | SSCDM1 | Non-celiac |
| --- | --- | --- | --- | --- |
| Asymptomatic | | 1/12 (8.3) | 13/34 (38.2) | 21/40 (52.5) |
| Symptoms related to iron deficiency | **≥1 iron deficiency–related symptom** | 5/12 (41.7) | 18/34 (52.9) | 10/40 (25) |
|  | **Fatigue / adinamia** | 4/12 (33.3) | 15/34 (44.1) | 9/40 (22.5) |
|  | **Hair loss** | 2/12 (16.7) | 1/34 (10) | 4/40 (10) |
|  | **Brittle nails** | 1/12 (8.3) | 9/34 (26.5) | 5/40 (12.5) |
|  | **Angular cheilitis** | 1/12 (8.3) | 0 (0) | 4/40 (10) |
|  | **Palpitations** | 0 (0) | 1/34 (2.9) | 0 (0) |
|  | **Palmar and plantar hyperhidrosis** | 0 (0) | 1/34 (2.9) | 0 (0) |
| Dyspepsia | **≥1 dyspepsia symptom** | 2/12 (16.7) | 4/34 (11.8) | 8/40 (20) |
|  | **Postprandial bloating** | 2/12 (16.7) | 3/34 (8.8) | 8/40 (20) |
|  | **Abdominal bloating / meteorism** | 1/12 (8.3) | 1/34 (2.9) | 5/40 (12.5) |
|  | **Flatulence** | 1/12 (8.3) | 0 (0) | 0 (0) |
|  | **Borborygmi** | 0 (0) | 3/34 (8.8) | 4/40 (10) |
| Non-dyspeptic gastrointestinal symptoms | **Diarrhea** | 1/12 (8.3) | 0 (0) | 3/40 (7.5) |
|  | **Constipation** | 0 (0) | 0 (0) | 2/40 (5) |
| Other symptoms | **Headache** | 0 (0) | 2/34 (5.9) | 1/40 (2.5) |
|  | **Rough tongue / atrophic glossitis** | 1/12 (8.3) | 0 (0) | 0 (0) |
|  | **Nausea** | 1/12 (8.3) | 1/34 (2.9) | 0 (0) |
|  | **Rhinosinusitis** | 0 (0) | 1/34 (2.9) | 1/40 (2.5) |

Postprandial bloating, meteorism, flatulence, and borborygmi were grouped and analyzed under a single dyspepsia category. ≥1 iron deficiency–related symptom was defined as the presence of at least one of the following: fatigue/adinamia, hair loss, brittle nails, angular cheilitis, palpitations, or palmar/plantar hyperhidrosis. ≥1 dyspepsia symptom was defined as the presence of at least one of the following: postprandial bloating, abdominal bloating/meteorism, flatulence, or borborygmi. Percentages have been rounded per convention. Variables are expressed as numbers and percentages. Abbreviations: SSCDM1, Suspected Seronegative Celiac Disease at Marsh 1.
